# Correlation Analysis of Serum Vitamin D Levels and Postoperative Cognitive Disorder in Elderly Patients With Gastrointestinal Tumor

**DOI:** 10.3389/fpsyt.2022.893309

**Published:** 2022-04-15

**Authors:** Jialei Zhang, Xiaoling Zhang, Yongyan Yang, Jun Zhao, Yonghao Yu

**Affiliations:** ^1^Department of Anesthesiology, Tianjin Medical University General Hospital, Tianjin, China; ^2^Department of Anesthesiology, Changzhi People's Hospital Affiliated to Shanxi Medical University, Changzhi, China; ^3^Department of Oncology, Changzhi People's Hospital Affiliated to Shanxi Medical University, Changzhi, China

**Keywords:** elderly population, perioperative, vitamin D, GSH, POCD

## Abstract

**Purpose:**

Vitamin D prevents hypocalcaemia, osteoporosis, and infections, among other problems, and is involved in the prevention and treatment of cardiovascular and neurological diseases. Recently, vitamin D was shown to improve cognitive dysfunction caused by Alzheimer's disease and vascular dementia. This study aims to explore the correlation between preoperative serum vitamin D and postoperative cognitive disorder (POCD) occurrence in elderly patients with gastrointestinal tumors to guide perioperative medication use and promote early patient recovery.

**Methods:**

This study recruited 238 elderly patients (65 ≤ age ≤ 85) who underwent gastrointestinal tumor surgery; 117 cases were enrolled, and 55 controls of the same age and education level as the cases were included. Blood samples were taken preoperatively and at 7, 15, 30, and 90 days postoperatively, and plasma vitamin D (25OH-D3) and glutathione (GSH) was measured. Different from the previous diagnosis of POCD was obtained by telephone interview through Cognitive Status Modified Telephone Interview (TICS-m), mainly for memory impairment, a series of neuropsychological tests was used to evaluate cognitive function, Picture Recollect Test, Stroop Color-word Test, and Digit Symbol Substitution Test were used to comprehensively evaluate the three domains of cognitive function of patients, namely memory, attention and information processing ability. All neuropsychiatric assessments were performed at the bedside and completed face-to-face by the assessment staff and the patient.

**Results:**

A total of 65.8% (77/117) of elderly patients undergoing gastrointestinal tumor surgery had preoperative vitamin D deficiency (serum 25OH-D concentration < 12 ng/ml), of whom 46.7% (36/77, 7 days after surgery), 31.2% (24/77, 15 days after surgery), 15.6% (12/77, 30 days after surgery), and 9% (7/77, 90 days after surgery) of patients developed POCD; 7.5% (3/40) of patients without vitamin D deficiency developed PNDs, which was detected only on the 7th day after surgery.

**Conclusions:**

Vitamin D deficiency can increase neurocognitive disorder risk in elderly patients during the perioperative period, possibly because low vitamin D levels cannot effectively inhibit the postoperative oxidative stress increase.

**Trial Registration:**

This experiment was approved and registered by the China Clinical Trial Registration Center, registration number ChiCTR2100046900 (30/05/2021).

## Key Summary Points

- **Aim:** To explore the correlation between vitamin D levels in elderly patients and the occurrence of postoperative cognitive disorder.
- **Findings:** The elderly are prone to vitamin D deficiency, and preoperative vitamin D deficiency is prone to postoperative cognitive disorder.
- **Message:** Low levels of vitamin D in elderly patients are associated with the occurrence of postoperative cognitive disorder.

## Introduction

The aging population has become a focus of society as a whole, and because of the continuous development of medical technology and continuous progress of science, the number of elderly patients undergoing surgery with anesthesia also increases every year. Although great progress has been made in surgical and anesthesia methods, the incidence of postoperative cognitive dysfunction in patients has indisputably increased. Studies have shown (1) that surgical factors (such as a long duration, a complex surgery, and the occurrence of postoperative complications) and anesthesia factors (such as the use of anesthetic drugs, occurrence of hypoxia and hypoperfusion caused by controlled hypotension, and occurrence of postoperative anesthesia-related complications) are the main factors that lead to postoperative cognitive dysfunction. However, regardless of the type of surgery and anesthesia, advanced age can be regarded as an independent risk factor for cognitive dysfunction after surgical anesthesia (2, 3), which merits close attention by medical workers.

Vitamin D deficiency has become a public health problem affecting the world, especially the elderly, and this problem is becoming more and more serious (4). China has conducted two general surveys on the vitamin D content of the elderly (5, 6), and the results show that up to 90% of the elderly lack vitamin D. Studies have shown that (7–9), as a neuroprotective hormone, vitamin D can pass through the blood-brain barrier, regulate the expression of key enzymes in neurotransmitter synthesis and metabolism in the brain, and participate in a variety of brains activity including neurotrophic, neuroimmunological regulation, and neurotransmission, thereby playing a certain protective effect on the central nervous system. The lack of vitamin D may be closely related to the occurrence of neuropsychiatric diseases such as Parkinson's disease, schizophrenia, and depression (10, 11).

Postoperative Cognitive Disorder (POCD) (12) refers to the disturbance of brain function activity after surgical anesthesia in patients without mental disorders before surgery, resulting in different degrees of disorder activity in consciousness, cognition, orientation, thinking, memory, and sleep. It is a reversible and fluctuating acute mental disorder syndrome, commonly known as postoperative mental disorder, postoperative delirium, etc., and is one of the important postoperative complications. With ongoing developments in medicine and improvements in quality of life, the average life expectancy of human beings has been extended, and an increasing number of elderly patients undergo surgical procedures each year. Although the techniques of surgery and anesthesia management are constantly improving, the incidence of POCD is increasing, and this is a problem that cannot be ignored. Once a patient develops POCD, not only are the effect of surgical treatment and recovery after anesthesia surgery affected but so are the workload and mental stress of the surgical and anesthesia medical staff. This study used long-term dynamic monitoring of perioperative serum vitamin D levels in patients with gastrointestinal tumors and long-term observation of patients' cognitive function to determine the correlation between vitamin D level and POCD and to present a comparative discussion.

## Experimental Procedures

### Study Design

This study is a single-center prospective cohort study. By comparing the effects of preoperative serum vitamin D levels on the occurrence of POCD in olderly patients undergoing gastrointestinal tumor surgery under intravenous inhalation combined with general anesthesia, we explored the correlation between serum vitamin D level and the occurrence of POCD in elderly patients. All subjects signed an informed consent form. This experiment was approved and registered by the China Clinical Trial Registration Center, registration number ChiCTR2100046900.

### Patients

The inclusion criteria were as follows: men and women aged 65-85 years old; American Society of Anesthesiologists (ASA) classification I-III; years of education ≥ 8 years; and no abnormal liver or kidney function.

The exclusion criteria were as follows: patients with severe respiratory and circulatory diseases, cognitive dysfunction, history of brain trauma and craniocerebral surgery or history of cerebral infarction; patients who have taken medical drugs containing vitamin D and folic acid in the past 2 months; and patients with a history of severe drug allergy.

The subjects in the control group were all patient spouses or siblings, and the inclusion and exclusion criteria were the same as those of the patients. No relevant surgery was performed on the control subjects. The controls and patients underwent neuropsychological testing at the same time. The purpose of the control group was to provide the standard deviation of the two cognitive tests of normal people and to eliminate the learning effect produced by the calculation test patients when they performed multiple repeated cognitive tests.

### Data Collection

The researchers responsible for patient data collection underwent thorough training before the start of the study. Data collection was only carried out after obtaining written informed consent. Basic data included demographic parameters (age, sex, height, weight, year of education), preoperative diagnosis, preoperative ASA classification, and coexisting diseases (hypertension, coronary heart disease, diabetes, chronic obstructive pulmonary disease, stroke, etc.), smoking history, drinking history, surgical history, preoperative neuropsychological test scores, preoperative serum vitamin D level and glutathione (GSH) content. Blood samples were collected at the bedside and sent to the Experimental Center of Changzhi People's Hospital for serum 25-hydroxyvitamin D and reduced glutathione concentration determination.

Intraoperative data included operation type, anesthesia time, operation time, type and dosage of anesthetic drugs, and bleeding volume. The depth of anesthesia was monitored, and the bispectral index (BIS) of all patients was maintained at 50~60.Intraoperative data were recorded by the anaesthesiologist following the operation. Basic data and postoperative data were the responsibility of the anaesthesiologist who was not involved in the operation and perioperative care. The two did not communicate on the patient's intraoperative and postoperative conditions. The patients and data collectors were not aware of the grouping of patients.

Postoperative data included the occurrence of complications within 30 days after surgery, the length of postoperative hospital stay, the 30-day mortality rate after surgery, the scores for various neuropsychological tests for patients at 7, 15, 30, and 90 days after surgery, serum vitamin D level and reduced GSH content.

All neuropsychiatric assessments were performed at the bedside and completed face-to-face by the assessment staff and the patient.

The researchers conducting the neuropsychological evaluations received specialized training in neuropsychological test management prior to the study. The neuropsychological tests included the following: the Mini Mental State Examination (MMSE), Digit Symbol Substitution Test (DSST), Stroop color-word test, and picture recollect test. Before surgery, all patients who met the inclusion criteria were tested by MMSE to identify whether cognitive dysfunction already existed. Studies have shown that (13), MMSE evaluates the cognitive function of elderly patients with a sensitivity of 87% and a specificity of 82%, so the reliability and reliability are high, and it is one of the most influential screening tools for cognitive function changes. Since the MMSE score is closely related to the level of education, the preoperative screening should be adjusted according to the level of education: 17 points for the illiterate group (no education); 20 points for the primary group (education years ≤ 6 years); the middle school group (education years 7~9 years) had 22 points, and the University group (education years ≥ 1 year) had 24 points. Patients with scores lower than the above criteria were excluded. After passing the MMSE test, they were included in the study. The DSST, Stroop color-word test and picture recollect test were performed preoperatively and 7, 15, 30, and 90 days postoperatively.

The diagnosis of POCD was based on the International Postoperative Cognitive Dysfunction Research Group (ISPOCD) (3). The diagnostic criterion was calculated as follows:


$$\begin{array}{l} Z=\frac{\text{Δ}X-\text{Δ}Xc}{SD\left(\text{Δ}Xc\right)} \end{array}$$

ΔX is the difference between the change in one test scale before and after the experiment. The ΔXc of the control group was also calculated, and the two values were subtracted and then divided by the standard deviation of ΔXc to obtain the *Z*-value. If there were multiple scales, the Zcombined value was calculated by determining the sum of the *Z*-values of each scale and dividing by the standard deviation of the Z-value of the control group. The calculation formula is:


$$\begin{array}{l} \text{Zcombined}=\frac{\sum Za,b,c,d}{SD\left(\sum \text{Δ}Xca,b,c,d\right)} \end{array}$$

When there are two or more Z-values ≥ 1.96 on all scales, or one scale Z-value ≥ 1.96 on all scales and a Zcombined ≥1.96 at the same time, POCD is diagnosed. This diagnostic method excludes the learning effect of repeated measurements and errors caused by measurements at different times and has good sensitivity and specificity.

Enzyme-linked immunosorbent assay (ELISA) was used to determine the levels of vitamin D and GSH in the blood. According to the literature (14), vitamin D levels are defined as follows: adequate: >20 ng/ml; sufficient: 12-20 ng/ml; deficient: <12 ng/ml.

### Statistical Analysis

#### Sample Size Estimation

We used a cross-sectional study on the correlation coefficient to calculate the sample size. Previous studies have shown that older age is moderately related to POCD (15–17). We assumed that low serum levels of vitamin D and POCD are at least correlated, setting significance (two-sided) at 0.05, power set at 0.8, and the effect size (r) = 0.3, dropouts = 0.20, the required sample size is 107 cases.

#### Outcome Analyses

Patients were grouped according to whether the vitamin D deficiency was present before surgery and whether they developed POCD after surgery. IBM SPSS Statistics 26.0, which is produced by SPSS, Inc., was used for data analysis. Measurement data are expressed as the mean ± standard deviation (*x* ± s), count data are expressed as the median and interquartile range, and classification data are expressed as quantity and percentage. One-way analysis of variance (one-way ANOVA) was used for comparisons between the two groups. The Shapiro-Wilk normality test was used to detect whether each group of data was normally distributed, and comparisons between the two groups was performed by *t*-test (normally distributed data) and Welch's correction test (nonpositive distribution data); categorical variables were analyzed by the chi-square test, continuity-adjusted chi-square test, or Fisher's exact test; and multiple comparisons were analyzed by the least significant difference (LSD) method. *P* < 0.05 was considered statistically significant.

## Results

### Patient Recruitment

**Figure 1:**
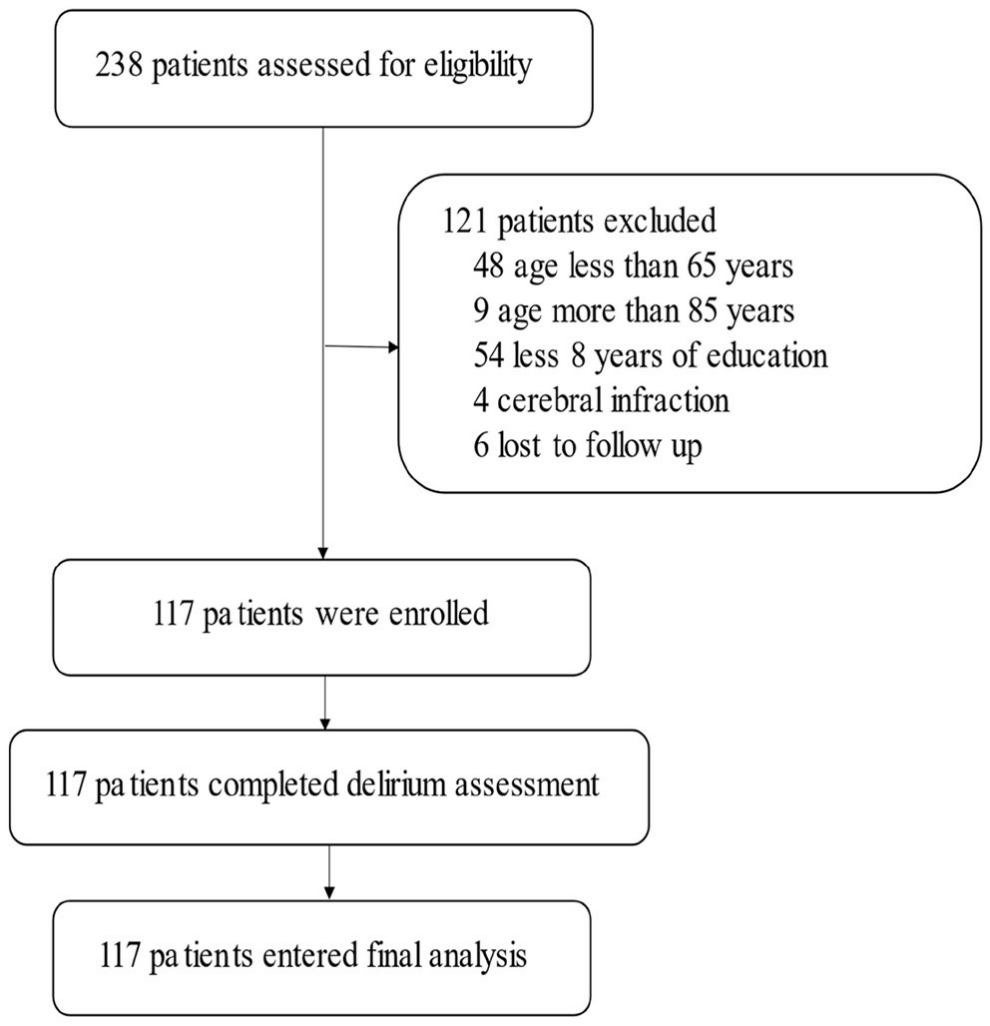
Flow chart of the study.

From October 1, 2020, to March 30, 2021, 238 cases of gastrointestinal tumor surgery occurred in our hospital; 121 cases were excluded, and 117 cases were included (Figure 1; Table 1). At the same time, 55 close relatives of patients were included in the control group and completed the same tests. There was no significant difference between the experimental group and the control group in terms of age (*P* = 0.085), body mass index (BMI) (*P* = 0.982), or education level (*P* = 0.414) (Table 2).

**Table 1:** Demographic and baseline characteristics.

	**All patients *n* = 117**	**VD deficiency *n* = 77**	**No VD deficiency *n* = 40**	***P*-value**
Age, year	71.08 ± 3.62	71.36 ± 3.63	70.57 ± 3.65	0.27
Male, *n*(%)	83(70.9)	50(64.9)	33(82.5)	0.055
BMI, kg/m^2^	23.57 ± 2.97	23.83 ± 3.19	23.07 ± 2.42	0.19
**ASA classification**, ***n*****(%)**				
I	24(20.5)	15(19.5)	9(22.5)	
II	81(69.2)	56(72.7)	25(62.5)	
III	12(10.3)	6(7.8)	6(15)	
Educational level, year	9.17 ± 1.74	9.23 ± 1.73	9.09 ± 1.77	0.59
Chronic smoking, *n* (%)	24(20.5)	13(16.9)	11(27.5)	0.228
Alcoholism, *n* (%)	9(7.7)	6(7.8)	3(7.5)	0.955
**Comorbidity**, ***n*** **(%)**				
Hypertension	70(59.8)	46(59.7)	24(60)	0.978
Diabetes mellitus	36(30.8)	25(32.5)	11(27.5)	0.675
Coronary heart disease	12(10.3)	6(7.8)	6(15)	0.335
Chronic lung diseases	7(6.0)	5(6.5)	2(5)	0.747
History of surgery, *n* (%)	14(11.9)	9(11.7)	5(8)	0.898
preoperative MMSE, score	27.05 ± 1.17	27.15 ± 1.27	27.05 ± 1.12	0.67
Vitamin D concentration, ng/ml	11.91 ± 3.67	9.59 ± 1.64	16.04 ± 3.27	<0.001
**Vitamin D level**, ***n*****(%)**				
Adequate	6(5.1)	–	6(15)	
Insufficient	34(29.1)	–	34(85)	
Deficient	77(65.8)	77(100)	–	
Serum GSH concentration, ug/ml	330.25 ± 16.49	336.46 ± 14.15	340.69 ± 19.18	0.179

**Table 2:** Baseline and follow-up data of control subjects and patients.

	**Control subjects (*n* = 55)**	**All patients (*n* = 117)**	***P-*value**
Age, year	71.91 ± 3.61	71.08 ± 3.62	0.165
BMI, kg/m^2^	24.08 ± 2.12	23.57 ± 2.97	0.252
Male gender	42(76.4)	83(70.9)	0.457
Educational level, year	9.07 ± 1.69	9.17 ± 1.74	0.728
MMSE, score	27.41 ± 1.27	27.05 ± 1.17	0.064

### Perioperative Outcome

Comparing patients with VD deficiency and patients without VD deficiency, there was no significant difference between the two in the use of anesthetic drugs, anesthesia time, operation time, blood loss and complications within 30 days after surgery (*P* > 0.05). Compared with non-POCD patients, POCD patients had more postoperative complications (*P* = 0.009) and longer hospital stay (*P* = 0.030) (Table 3).

**Table 3:** Perioperative variables.

	**All patients *n* = 117**	**VD deficiency *n* = 77**	**No VD deficiency *n* = 40**	***P*-value**	**POCD *n* = 43**	**No POCD *n* = 74**	***P*-value**
Duration of anesthesia, min	251.12 ± 20.63	250.44 ± 20.03	252.42 ± 21.93	0.624	250.91 ± 19.20	250.44 ± 21.54	0.933
Duration of surgery, min	205.55 ± 19.39	206.28 ± 19.80	204.12 ± 18.73	0.573	209.44 ± 20.03	200.14 ± 19.03	0.802
**Anaesthetic drugs**							
Propofol	500.89 ± 57.18	501.48 ± 57.02	499.75 ± 58.19	0.882	495.42 ± 58.37	504.07 ± 56.63	0.433
Remifentanil	1.20 ± 0.12	1.21 ± 0.12	1.19 ± 0.11	0.519	1.19 ± 0.13	1.21 ± 0.11	0.626
Sevoflurane	43.92 ± 8.61	44.23 ± 8.44	43.32 ± 9.00	0.587	45.30 ± 9.24	42.53 ± 8.84	0.681
Estimated blood loss	109.32 ± 12.92	108.45 ± 13.33	111.03 ± 12.08	0.313	109.27.44 ±10.91	101.44 ± 14.03	0.614
Complications within 30 days	19(16.2)	16(20.8)	3(7.5)	0.111	12(27.9)	7(9.4)	0.009
Mortality within 30 days Length of stay in hospital	0(0.0) 15.01 ± 1.65	0(0.0) 15.25 ± 1.75	0(0.0) 14.88 ± 1.59	0.257	0(0.0) 15.44 ± 1.50	0(0.0) 14.76 ± 1.69	0.030

### PND Statistics

**Figure 2:**
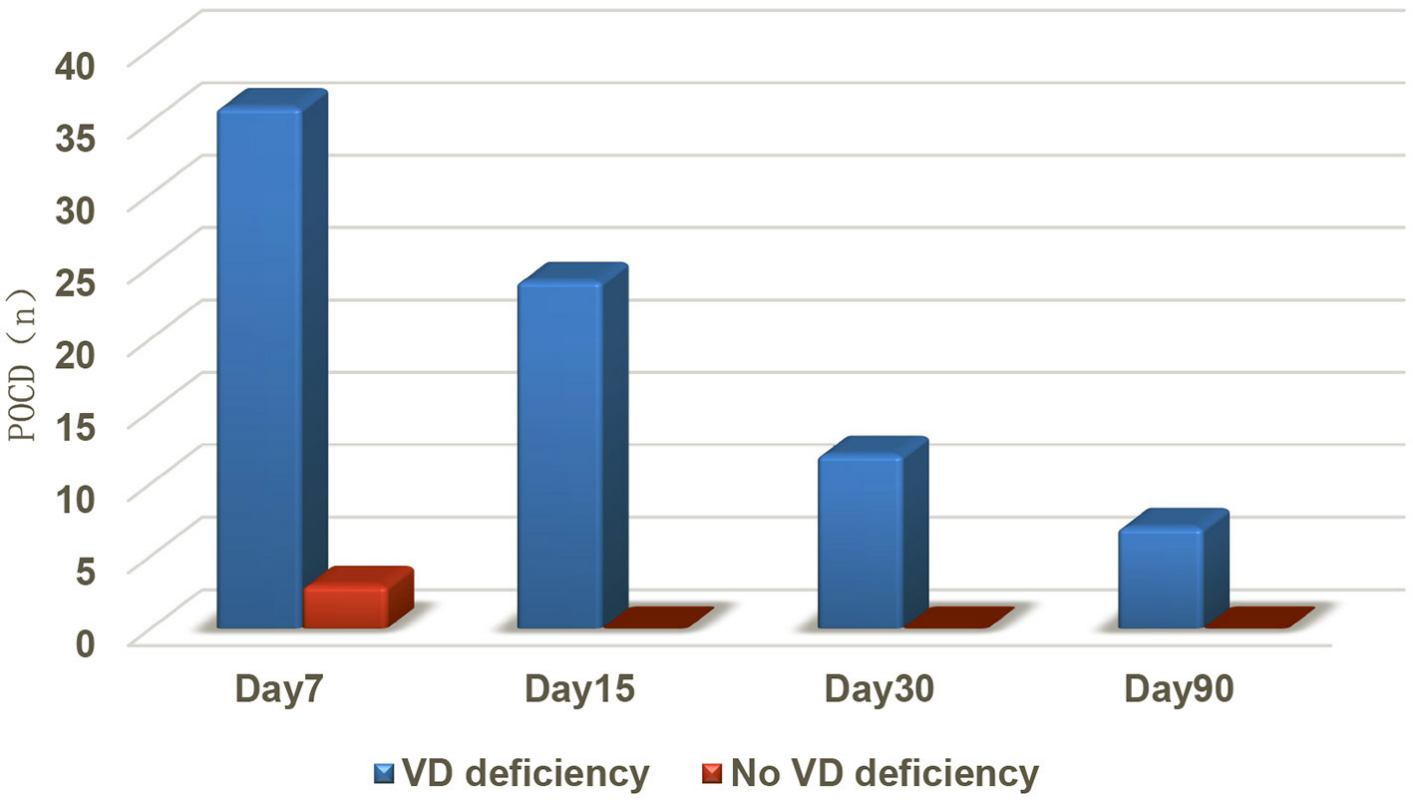
Comparison of the number of patients with POCD between the two groups.

This study showed that the number of patients who developed POCD after surgical anesthesia was 39 at 7 days, 24 at 15 days, 12 at 30 days, and 7 at 90 days. Among them, the incidence of POCD in patients with vitamin D deficiency (77/117) was 46.7% (36/77) 7 days after surgery, 31.2% (24/77, four new cases) 15 days after surgery, 15.6% (12/77) 30 days after surgery, and 9% (7/77) 90 days after surgery, which was much higher than that in patients without vitamin D deficiency (40/117), the incidence of POCD in non-deficient patients was 7.5% (3/40, present only 7 days after surgery) (*P* < 0.000), showing that there was a correlation between preoperative vitamin D deficiency and POCD, and that higher preoperative levels of serum vitamin D (25OH-D) effectively reduced the occurrence of POCD (Figure 2; Table 4).

**Table 4:** PND analysis.

	**POCD *n* = 43**	**No POCD *n* = 74**	***P*-value**
VD deficiency	40(93.02)	37(50.00)	*P* < 0.001
No VD deficiency	3(6.98)	37(50.00)	
Vitamin D concentration, ng/ml	9.90 ± 1.71	13.03 ± 4.04	*P* < 0.001
**Z-score, score**			
day 7	2.94(2.81,3.07)	0.98(0.94,1.02)	*P* < 0.001
day 15	2.54(2.55,2.82)	1.11(1.10,1.18)	*P* < 0.001
day 30	2.46(2.15,2.54)	0.76(0.84,0.91)	*P* < 0.001
day 90	2.44(1.98,2.37)	0.71(0.62,0.67)	*P* < 0.001

### Changes in Serum Vitamin D and GSH

**Figure 3:**
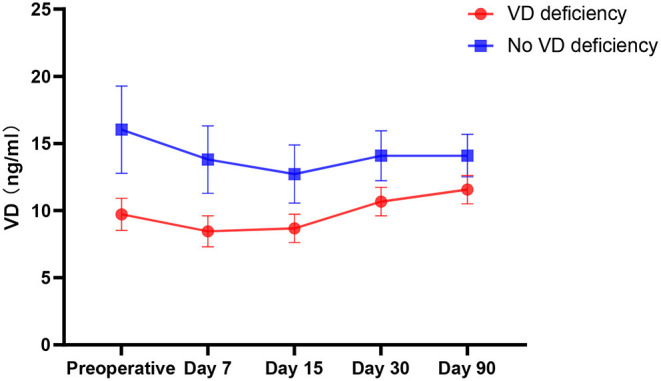
Trends in Vitamin D.

This study also found that 65.8% (77/117) of patients had vitamin D deficiency before surgery, which is consistent with the findings of previous related studies (5, 18). Moreover, after surgical anesthesia, the serum levels of 25OH-D (vitamin D) of all patients decreased significantly compared with those before surgery (*P* < 0.001) (Figure 3), reaching the lowest value 15 days after surgery then rising gradually. Patients with vitamin D deficiency had a greater rate of increase than those without vitamin D deficiency.

**Figure 4:**
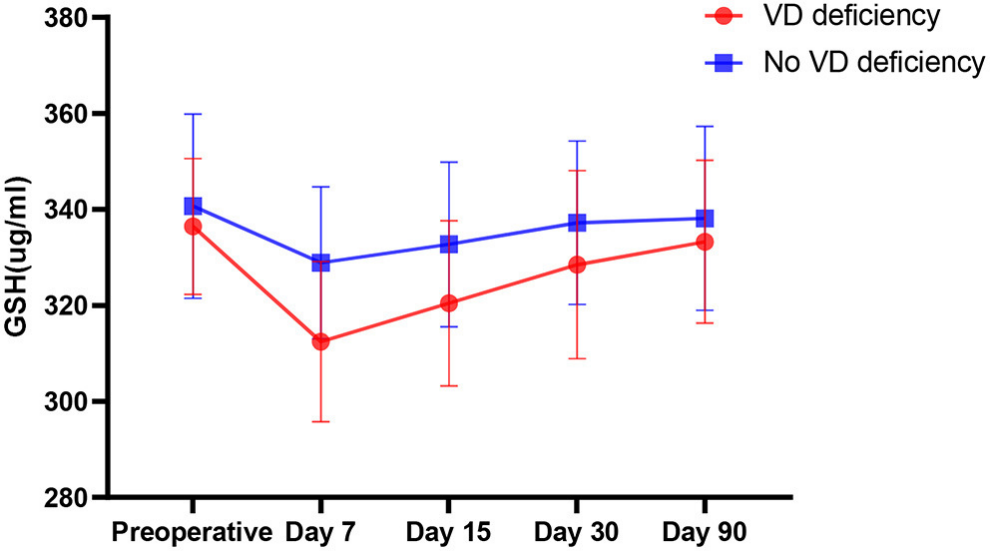
Comparison of the change trend of serum GSH in patients with VD deficiency and non-VD deficiency before operation.

This study also analyzed the trend of changes in serum GSH, found that there was no significant difference between patients with vitamin D deficiency and those without deficiency before surgery (*P* = 0.179). After surgical anesthesia, GSH and vitamin D decreased significantly, and the decrease in vitamin D deficient patients was greater than that in patients who were not vitamin D deficient (*P* < 0.000) (Figure 4).

### Analysis of the Correlation Between Vitamin D Level and POCD

We use rank correlation, also called rank correlation, to analyze the correlation between preoperative vitamin D levels and POCD to describe the degree and direction of the correlation between the two. The results showed that the Spearman correlation coefficient was −0.417 and *P* < 0.001 when the two variables were at a two-sided confidence level of 0.01, indicating that the two variables are correlated and negatively correlated, that is, the higher the vitamin D level, the lower the probability of POCD.

### Confounding Factor Analysis

This study used single factor logistic regression analysis to analyse count data [sex, smoking, drinking, hypertension, diabetes, coronary heart disease, chronic obstructive pulmonary disease (COPD), history of surgery, ASA classification] and measurement data (vitamin D content, age, operation time, anesthesia). Time, education time, BMI, intraoperative blood loss, and anesthetic dosage were analyzed separately. The analysis showed that preoperative vitamin D content (deficiency, <12 ng/ml) was related to the occurrence of POCD (*P* < 0.000), and two potential confounding factors were identified: sex (*P* = 0.034) and age (*P* = 0.048). Further analysis concluded that non-vitamin D deficient patients had a reduced occurrence of POCD (OR: 0.075, 95% CI: 0.021-0.264, *P* < 0.000), and vitamin D deficient patients had an increased risk of POCD (OR: 13.333, 95% CI: 3.787-46.942, *P* < 0.000).

## Discussion

Due to the advancement of medical technology, the improvement of public health and the implementation of social welfare policies, the proportion of the elderly in the global population has accelerated, and the problem of population aging has become the focus of attention of the whole society (19). At the 14th Vitamin D Nutrition Guidelines Symposium, some researchers proposed that half of the elderly in North America have vitamin D deficiency, and in other countries around the world, 2/3 of the elderly have the same problem (20). In recent years, researchers have begun to study the relationship between vitamin D deficiency and cognitive dysfunction in the elderly. Although a large number of studies have shown that vitamin D has a positive effect on brain function (21–23), but some studies have raised objections to this (24–26), they believe that the incidence of vitamin D on cerebrovascular adverse events is no different from the placebo group.

This study aims to better discover and explore the relationship between low levels of vitamin D and POCD in elderly patients through long-term clinical trials, so as to guide the direction of clinical perioperative medication and animal experiments. As far as we know, this is the first time that such a long period of perioperative vitamin D level monitoring has been carried out, and study the effect of surgical anesthesia on the vitamin D concentration in the body, as well as the analysis of the relationship between preoperative vitamin D level and POCD.

Current investigations on the correlation of cognitive decline lack clinical case diagnosis and can only rely on functional scales. For patients after surgery, the diagnosis of POCD was obtained through telephone interviews via the Telephone Interview for Cognitive Status-modified (TICS-m), which is mainly used for the evaluation of cognitive impairment, namely, memory impairment, but has poorer mental flexibility and function evaluation. Compared with the method adopted in this experiment, it is more sensitive to diagnosis, and the specificity is poor. In this study, we used color-word separation experiments, digital symbol conversion experiments, and picture memory experiments on all patients, including those in the control group, to evaluate cognitive status. These experiments included the possible influence of surgery and anesthesia on areas such as memory, concentration, graphic and visual memory, writing flexibility, judgment, and brain speed. The diagnosis is based on the International Study of POCD1 (ISPOCD1). This diagnostic method excludes the learning effect of repeated measurement and the error caused by measurement at different times and has good sensitivity and specificity.

Our research results show that among 117 elderly patients who underwent gastrointestinal tumor surgery, 65.8% (77/117) were vitamin D deficient, and of these, 46.7% (36/77, 7 days after surgery) and 31.2% (24/77, 15 days after surgery), 15.6% (12/77, 30 days after surgery), and 9% (7/77, 90 days after surgery) of patients developed PND, which is much higher than the percentage of patients without vitamin D deficiency (3/40, 7.5%, and only exists 7 days after surgery) (*P* < 0.000). This suggests that low levels of serum 25(OH)D may be related to POCD, and higher concentrations of serum 25(OH)D can reduce the incidence of POCD.

After statistical analysis of other confounding factors that may cause POCD, we found that sex (*P* = 0.034) and age (*P* = 0.048) were also related to the occurrence of POCD. However, after correcting for these confounding factors (logistic regression), sex (*P* = 0.080, 95% CI: 0.906-5.664) and age (*P* = 0.219, 95% CI: 0.953-1.232) were excluded. Vitamin D level was still the key factor affecting the occurrence of POCD (P < 0.000, 95% CI: 0.565-0.847).

The study also found that 65.8% (77/117) of patients were vitamin D deficient before surgery and that after surgery anesthesia, the serum 25OH-D (vitamin D) content of all patients decreased significantly compared with that before surgery (*P* < 0.001); the lowest value was reached 15 days after surgery. It is speculated that oxidative stress occurs in the human body after surgery, which depletes the body of vitamin D. At the same time, gastrointestinal function was not restored after the operation, and vitamin D in food could not be well absorbed. Moreover, long-term bed rest after surgery and insufficient sun exposure may reduce the synthesis of vitamin D in the body. After 15 days after surgery, vitamin D levels began to rise gradually, and patients with vitamin D deficiency had a greater rate of increase than non-deficient patients. It is speculated that on the one hand, the patient's gastrointestinal function recovers after the operation and can absorb nutrients in the diet well; on the other hand, whether there is a “ceiling” effect in the vitamin D absorption process in elderly patients—that is, if the patient is presently vitamin D deficiency, the rate of increase will increase rapidly after supplementation, and if the patient is in a non-deficient state, the rate of increase will be slower as the patient cannot fully absorb and synthesize vitamin D in food. This requires further research.

Studies have shown that the antioxidant effect of vitamin D is achieved, on the one hand, by inhibiting the production of nitric oxide synthase (27) and, on the other hand, by activating the inherent antioxidant pathway to enhance the content of γ-glutamyl transpeptidase, thus increasing the level of glutathione (28). As a natural antioxidant, glutathione can protect the integrity of oligodendrocytes and nerve conduction pathways and play a vital role in the transmission of nerve information. Therefore, we also carried out simultaneous detection of GSH in the body. According to the experimental results, compared with preoperative levels, serum GSH after surgical anesthesia also decreased to varying degrees, and the levels of preoperative vitamin D deficient patients decreased more significantly than those of patients without a vitamin D deficiency (*P* < 0.000). This also indirectly proves that surgical anesthesia causes oxidative stress in patients, and preoperative vitamin D deficient patients have more severe oxidative stress. At the same time, studies have confirmed (29) that the decline in serum GSH may cause cognitive dysfunction in elderly patients. It is speculated that this may also be one of the reasons for the occurrence of POCD due to vitamin D deficiency.

Although this study found differences in the effects of serum vitamin D concentrations on POCD, there are still some shortcomings. First, studies have shown that there is a correlation between the patient education levels and the occurrence of postoperative cognitive dysfunction (13). In order to eliminate this interfering factor, this study set a higher level of education time (≥8 years). The setting excluded many elderly patients from the scope of this experiment, which is the biggest regret of the experiment. Second, the experimental cases were mainly collected in winter, and the vitamin D levels of elderly patients are generally low in this season, and the patients all underwent gastrointestinal tumor surgery, so they spent a long time in bed, which decreased sun exposure and affected the postoperative vitamin D concentration. Third, the patients all received radiotherapy and chemotherapy after the operation, and the radiotherapy and chemotherapy drugs used may have had some influence on the experimental results. Fourth, there was a large difference in the number of males and females in the included cases, which makes it impossible to fully analyse the sex differences.

## Conclusion

Our results show that 65.8% of elderly patients undergoing gastrointestinal tumor surgery have vitamin D deficiencies and that 46.7% of them have neurocognitive disorders after surgery, the incidence of POCD is much higher than that of patients without vitamin D deficiency; moreover, a small number of patients still have cognitive dysfunction even 90 days after surgery. Thus, a correlation between low levels of serum vitamin D and the occurrence of POCD in elderly patients is thought to exist, this may be related to the decrease in antioxidant stress in the body caused by vitamin D deficiency. The underlying mechanism and whether vitamin D supplementation can reduce the occurrence of POCD requires further study.

## Data Availability Statement

The original contributions presented in the study are included in the article/supplementary files, further inquiries can be directed to the corresponding author/s.

## Ethics Statement

The studies involving human participants were reviewed and approved by Medical Ethics Committee of People's Hospital of Changzhi City. The patients/participants provided their written informed consent to participate in this study.

## Author Contributions

JZhan and YYu designed the study and wrote the manuscript. JZhan, XZ, YYa, and JZhao performed the experiments. JZhan and XZ analyzed the data. All authors contributed to the article and approved the submitted version.

## Conflict of Interest

The authors declare that the research was conducted in the absence of any commercial or financial relationships that could be construed as a potential conflict of interest.

## Publisher's Note

All claims expressed in this article are solely those of the authors and do not necessarily represent those of their affiliated organizations, or those of the publisher, the editors and the reviewers. Any product that may be evaluated in this article, or claim that may be made by its manufacturer, is not guaranteed or endorsed by the publisher.
